# Species-specific histological characterizations of renal tubules and collecting ducts in the kidneys of cats and dogs

**DOI:** 10.1371/journal.pone.0306479

**Published:** 2024-07-03

**Authors:** Shunnosuke Kira, Takashi Namba, Masaya Hiraishi, Teppei Nakamura, Yuki Otani, Yasuhiro Kon, Osamu Ichii

**Affiliations:** 1 Laboratory of Anatomy, Department of Basic Veterinary Sciences, Faculty of Veterinary Medicine, Hokkaido University, Sapporo, Hokkaido, Japan; 2 Laboratory of Laboratory Animal Science and Medicine, Department of Basic Veterinary Sciences, Faculty of Veterinary Medicine, Hokkaido University, Sapporo, Hokkaido, Japan; 3 One Health Research Center, Hokkaido University, Sapporo, Hokkaido, Japan; RWTH Aachen University Medical Faculty: Rheinisch-Westfalische Technische Hochschule Aachen Medizinische Fakultat, GERMANY

## Abstract

The histomorphological features of normal kidneys in cats and dogs have been revealed despite the high susceptibility of cats to tubulointerstitial damage. Herein, the histological characteristics of the two species were compared. Cytoplasmic lipid droplets (LDs) were abundant in the proximal convoluted tubules (PCTs) of cats aged 23–27 months but scarce in dogs aged 24–27 months. LDs were rarely observed in the distal tubules (DTs) and collecting ducts (CDs) of either species, as visualized by the expression of Tamm–Horsfall protein 1, calbindin-D28K, and aquaporin 2. The occupational area ratio of proximal tubules (PTs) in the renal cortex was higher, but that of DTs or CDs was significantly lower in adult cats than in dogs. Single PT epithelial cells were larger, but PCT, DT, and CD lumens were significantly narrower in adult cats than in dogs. Unlike adults, young cats at 6 months exhibited significantly abundant cytoplasmic LDs in proximal straight tubules, indicating lipid metabolism-related development. Histochemistry of the 21 lectins also revealed variations in glycosylation across different renal tubules and CDs in both species. Sodium-glucose cotransporter 2 was expressed only in PTs, excluding the proximal straight tubules with few LDs in adult cats or the PCTs of young cats and adult dogs. These findings are crucial for understanding species-specific characteristics of renal histomorphology and pathogenesis.

## Introduction

The mammalian kidney is derived from the metanephric blastema and ureteric bud during the fetal period, and each forms a nephron and collecting duct (CD). Nephrons include Bowman’s capsule, proximal tubules (PTs), ascending and descending loops of Henle (also referred to as attenuated tubules in veterinary histology), and distal tubules (DTs) following CD. Each segment of the nephron or CD has a region-specific function. Therefore, the expression of markers such as aquaporin 1 (AQP1) in PTs, Tamm–Horsfall protein 1 (THP1) in distal straight tubules (DSTs), calbindin-D28K (CD28K) in distal convoluted tubules (DCTs), and aquaporin 2 (AQP2) in CDs [1–3] has been used to identify each segment using histological methods. However, these marker detection methods cannot be applied to all animals because of species-specific differences in these molecules’ expression or amino acid sequences. In addition, as glycan expression shows nephron- or CD-specific patterns, lectin histochemistry has been used to identify each tubule in various species, including dogs and cats [4, 5]. Glycans contribute to the structure and organization of cell walls, extracellular matrices, and the proper folding and stability of glycoproteins [6]. Understanding histomorphological characteristics is crucial for clarifying renal function in each animal species.

The PT segment is the longest part of the nephron, and the reabsorption rate of Na^+^ reaches approximately 70% of the renal system in rat kidneys [7]. PTs are divided into three segments based on the type of epithelial cells in human and rat kidneys: S1, S2, and S3 segments [8, 9]. Similarly, cats have three distinct PT segments according to different staining patterns of periodic acid-Schiff (PAS) and immunohistochemistry (IHC) for AQP1 [2]. PT-specific expression has also been observed in kidney injury molecule-1, a representative marker of acute kidney injury (AKI) in humans [10]. Kidney injury molecule-1 is also a ligand for apoptosis inhibitor of macrophages (AIM), and AIM attached to debris is taken up by PT epithelial cells through phagocytosis [11]. Furthermore, sodium-glucose cotransporter 2 (SGLT2) is expressed specifically in PTs, and it participates in approximately 90% of glucose reabsorption from primary urine in humans [12]. SGLT2 inhibitors have been widely used to treat human diabetes, and their therapeutic effects on other diseases, including chronic kidney disease (CKD), have recently been demonstrated [13]. Thus, elucidating nephron-specific molecules could potentially lead to the development of diagnosis and therapy in human and veterinary medicine.

CKD prevalence in cats increases with age, ranging from 28% in cats over 12 years [14] to 81% in cats over 15-years-old [15]. Furthermore, approximately 51% of felids, including cats, have tubulointerstitial nephritis [16]. The scientific reasons remain unknown, but it has been suggested that several factors, such as low water intake, high incidence of urolithiasis, and AIM dysfunction, may be related to the high susceptibility of cats to CKD [17]. Another crucial species-specific characteristic is that cat kidneys contain abundant lipid droplets (LDs) in the epithelial cells of proximal convoluted tubules (PCTs) [18]. In general, lipids in the kidneys accumulate in the PTs, and high concentrations of albumin-bound long-chain saturated fatty acids promote tubular damage following interstitial fibrosis [19]. Furthermore, PT epithelial cells seem more sensitive to lipotoxicity than other cell populations in the kidney because of their need for higher energy levels, and energy production in them largely depends on lipolysis, unlike other nephron segments; lipid-induced mitochondrial damage may be particularly catastrophic for PCT epithelial cells [20–23].

This study aimed to clarify the histological characteristics of renal tubules and CDs by investigating the segment-specific expression of molecules, including SGLT2, and to compare the results between cats and dogs, which are representative companion animals. Furthermore, we focused on LD localization in cat PTs and lectin-binding patterns throughout renal tubules and CDs. The data obtained are essential for discussing species-specific renal morphology, function, and pathogenesis, especially the high susceptibility of felids to tubulointerstitial damage.

## Materials & methods

### Sample information

All samples were obtained from cadavers euthanized during other experiments. The kidneys were collected from healthy cats (mixed breed, intact adult males, n = 4, 23–27 months old; intact young males, n = 4, 6 months old) and dogs (beagle, intact males, n = 4, 24–27 months old). Adult cats and dogs were euthanized for use in the experiments, and this study was approved by the Institutional Animal Care and Use Committee of the Faculty of Veterinary Medicine, Hokkaido University (approval nos. 14–0054 and 20–0081, respectively) [24]. Experiments were performed in accordance with the Guidelines for Animal Experimentation of Hokkaido University, Japan. Additionally, we used kidney tissues from young cats euthanized in another experiment at Kitasato Institute (Saitama, Japan; provided by Dr. Nomoto) [25].

### Histological analysis

The collected kidneys were sliced to a thickness of approximately 5 mm (1 × 1 cm). The kidneys were fixed in 10% neutral buffered formalin for histological analysis and *in situ* hybridization (ISH) at 25°C for 3 d or 4% paraformaldehyde in 0.1 M phosphate buffer for IHC and lectin histochemistry at 4°C for 24 h. Fixed tissues were dehydrated using alcohol and embedded in paraffin. Histological sections 5-μm-thick for ISH or 2-μm-thick for other methods were prepared. For histological analysis, the sections were stained with PAS-hematoxylin (PAS-H).

### IHC and lectin histochemistry

IHC staining for THP1 (Santa Cruz, TX, USA) and AQP2 (Alpha Diagnostic Intl, TX, USA) was performed to identify DSTs and CDs, respectively. To identify DCTs, we referred to the localization of CD28K (Proteintech, Tokyo, Japan)-positive (^+^) reactions in humans [3]. Details of antigen retrieval, dilution, and antibodies used are listed in S1 Table. To characterize each segment based on glycan and lectin binding abilities, lectin histochemistry was performed, as shown in S2 Table.

The sections were then deparaffinized and rehydrated. Antigen retrieval was performed for IHC. Subsequently, the sections were soaked in methanol containing 0.3% H_2_O_2_ for 20 min at 25°C to block internal peroxidase activity. After washing three times in 10 mM phosphate-buffered saline (PBS, pH 7.4), the sections were incubated with a blocking serum for IHC or 1% bovine serum albumin in PBS for lectin histochemistry for 1 h at 25°C to block the non-specific reaction. Then, sections were incubated with primary antibodies or diluted biotinylated lectins overnight at 4°C. The sections were then washed three times with PBS. For IHC, sections were incubated with secondary antibodies for 30 min at 25°C and washed thrice in PBS. Consequently, the sections for IHC and lectin histochemistry were incubated with streptavidin-conjugated horseradish peroxidase (Nichirei; Tokyo, Japan) for 30 min at 25°C, washed three times in PBS, and the immune reaction was visualized with 3,3′-diaminobenzidine tetrahydrochloride in 0.05 M Tris-HCl buffer-H_2_O_2_ solution. Finally, IHC and lectin histochemical sections were stained with PASH and hematoxylin, respectively.

### Histomorphometry

Sections were visualized using PAS-H or IHC to evaluate PTs, DTs, and CDs. These images were converted to virtual slides using a Nano Zoomer 2.0 RS (Hamamatsu Photonics Co., Ltd., Hamamatsu, Japan), followed by NDP. View2 (Hamamatsu Photonics Co., Ltd.) was used for histomorphometry.

### Definition of whole and luminal areas in PT, DT, and CD

The area lined by the outer circumference of the tube or duct structures was defined as the entire area that lines the apex of the brush border, and the apical portion of the epithelial cells was defined as the luminal area.

### Occupation ratio of PT, DT, and CD in the unit area of the renal cortex

To measure the occupation ratio of PT, DT, and CD to the unit area, we used sections visualized by IHC for AQP2 counterstained with PAS-H (one section from each cat and dog). PTs and DTs were AQP2-negative (^-^), but the former possesses PAS^+^ brush borders. CD analysis revealed AQP2^+^ reactions. We randomly set 40 1 mm^2^ areas of the cortex and measured the whole area of each PT, DT, and CD, and the occupation ratio of each 1 mm^2^ area was expressed as a percentage.

### Size of a single epithelial cell ratio and the ratio of luminal area to the whole area in PTs, DTs, and CDs

As described in the calculation of the occupation ratio, the entire area of the PTs, DTs, and CDs was measured (four sections from each cat and dog, for 50 tubules or ducts). The luminal area was measured in the same tubule or duct and subtracted from the entire area. These values were divided by the number of nuclei to reflect the size of a single epithelial cell. The ratio of the luminal area to the entire luminal area was then calculated.

### Segmental definition of PTs in cat

Using PAS-H-stained sections, we defined four segments according to lipid droplet content in the cytoplasm of cat PT epithelial cells. Briefly, in the convoluted part, PCTs were semi-quantitatively divided into two segments: the segment in which the area of LDs occupied ≥50% or <50% cytoplasm area was defined as PCT^LD++^ or PCT^LD+^, respectively. In the medullary rays, proximal straight tubules (PSTs) were divided into two segments: the segment containing LDs in its cytoplasm was defined as PST^LD+^, and that containing few LDs was defined as PST^LD-^. We randomly selected 150 ducts for the PCT and PST and classified them into finer segments according to the LD content. These measurements were performed on adult and young cats (four sections per group).

### ISH

We used RNAscope to detect *SGLT2* in the kidneys of cats and dogs because we could not obtain clear IHC data for SGLT2 in a preliminary study of cat kidneys. Formalin-fixed paraffin-embedded tissue sections were deparaffinized in xylene, rehydrated in 100% ethanol, and finally dried completely. Sections were incubated in RNAscope hydrogen peroxide at 25°C for 10 min. These sections were incubated in RNAscope Target Retrieval Reagents (Advanced Cell Diagnostics, Inc.; Newark, CA, USA) for antigen retrieval at 95°C for 15 min. Sections were digested with RNAscope Protease Plus at 40°C for 30 min. After probe hybridization for 2 h at 40°C, amplification steps were performed as described in the RNAscope 2.5 HD Brown user manual. Subsequently, the sections were washed twice in RNAscope wash buffer, and positive reactions were visualized using the RNAscope 2.5 HD Reagent Kit (Brown). Finally, the sections were slightly stained with hematoxylin and blue in 0.02% NH_3_-water.

### Statistical analysis

The results are shown as the mean ± standard error and statistically analyzed in a non-parametric manner. Briefly, the significance between two groups (adult cats vs. adult dogs or adult cats vs. young cats) was analyzed using the Mann–Whitney *U*-test (*P* <0.05).

## Results

### Histological features of renal cortices in adult cats and dogs

**Fig 1:**
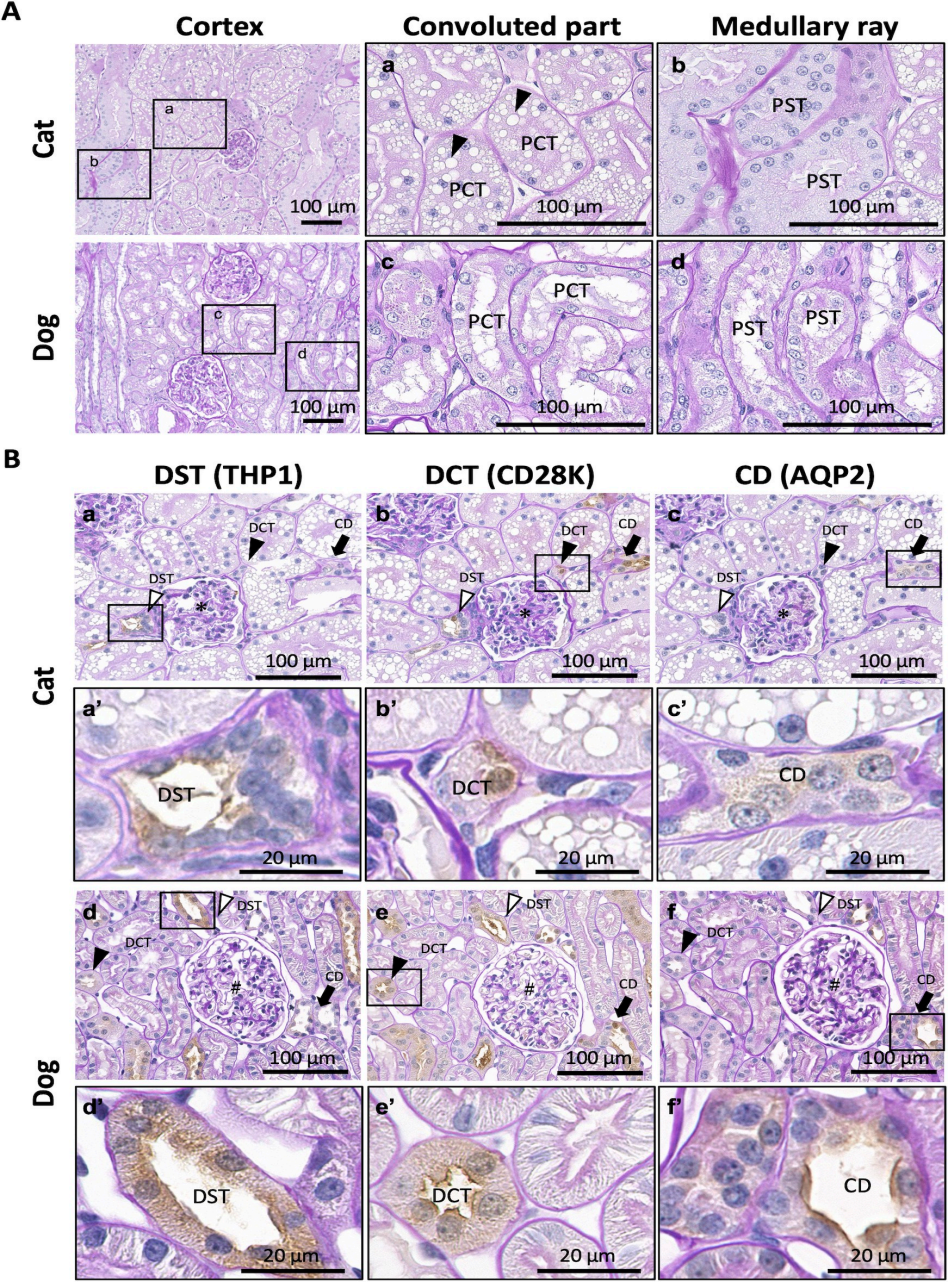
Histological features of renal cortices in adult cats and dogs. (A) Proximal tubules. Proximal convoluted tubules (PCTs) and proximal straight tubules (PSTs) are observed in the convoluted and medullary regions, respectively. Panels a–d magnified the squared areas in the left panels. Arrowheads denote abundant lipid droplets (LDs) in the cytoplasm of cat PCT epithelial cells. Periodic acid Schiff-hematoxylin (PAS-H) staining. (B) Distal tubules (DTs) and collecting ducts (CDs). The squared areas in panels a–f are magnified in panels a’–f’, serial sections for each animal. Asterisks and sharps indicate glomeruli (panels a–c and d–f, respectively). In both species, Tamm–Horsfall protein 1 (THP1) is positive in the distal straight tubules (DSTs; white arrowheads) but not in the distal convoluted tubules (DCTs; black arrowheads) and CDs (black arrows) (panels a, a’, d, and d’). Calbindin-D28K (CD28K) is positive for DSTs, DCTs, and CDs (panels b, b’, e, and e’). Aquaporin 2 (AQP2) is positive in CDs (black arrows) but not in DSTs and DCTs (panels c, c’, f, and f’). Immunohistochemistry was performed using PAS-H staining. Resolution of anatomical or histological images: 300 × 300 dpi.

Fig 1 shows the PAS-H-stained images of the renal cortices. In cats, the convoluted part included PCTs containing abundant LDs in the cytoplasm of the epithelial cells (Fig 1A–a). PSTs were observed in the medullary rays, while LDs were scarcely observed in the epithelial cells (Fig 1A–b). In dogs, PCTs and PSTs were found in the convoluted part and medullary ray, respectively, and both PT segments scarcely contained LDs and showed a larger luminal area and smaller cytoplasm of epithelial cells than in cats (Fig 1A–c, d).

Using IHC to detect renal tubule- and CD-specific proteins with normal PAS-H staining, it was possible to distinguish between DSTs, DCT, and CDs in cats and dogs (Fig 1B). Briefly, THP1^+^ staining was observed in the apical portion and cytoplasm of the DST and macular densa of cats and dogs (Fig 1B–a, d). In accordance with a previous report on human kidneys [3], we used CD28K as a DCT-specific marker. However, CD28K^+^ staining was observed in the epithelial cells of DSTs, DCTs, and CDs (Fig 1B–b, e). AQP2^+^ reactions were observed in the cytoplasm and apical portions of CDs in cats and dogs, respectively (Fig 1B–c, f). These reaction patterns were similar between both species; however, the sizes of the whole and luminal areas of the DSTs, DCTs, and CDs were larger in dogs than in cats (Table 1).

**Table 1:** Summary of positive immunohistochemistry in the kidneys of cats and dogs.

Structures		THP1		AQP2		CD28K	
		Cat	Dog	Cat	Dog	Cat	Dog
Renal corpuscle		–	–	–	–	–	–
Proximal tubule	PCT	–	–	–	–	–	–
	PST	–	–	–	–	–	–
Attenuated tubule		–	–	–	–	–	–
Distal tubule	DST	E-Ap, E-Cy	E-Ap, E-Cy	–	–	E-Cy	E-Ap
	DCT	–	–	–	–	E-Cy	E-Ap, E-Cy
Collecting duct	CCD	–	–	E-Cy	E-Ap	E-Cy	E-Cy
	OMCD	–	–	E-Ap, E-Cy	E-Cy	–	E-Cy
	IMCD	–	–	E-Cy	E-Cy	–	–
Interstitium, capillary		–	–	–	–	–	–

THP1, Tamm–Horsfall protein 1. CD28K, Calbindin-D28K. AQP2, Aquaporin 2. PCT, proximal convoluted tubule. PST, proximal straight tubule. DCT, distal convoluted tubule. DST, distal straight tubule. CCD, cortical collecting duct. OMCD, outer medullary collecting duct. IMCD, inner medullary collecting duct. E, epithelial cell. Cy, cytoplasm. Ap, apical portion. −, negative.

### Histomorphometric characteristics of each renal tubule and CD in the renal cortices of adult cats and dogs

**Fig 2:**
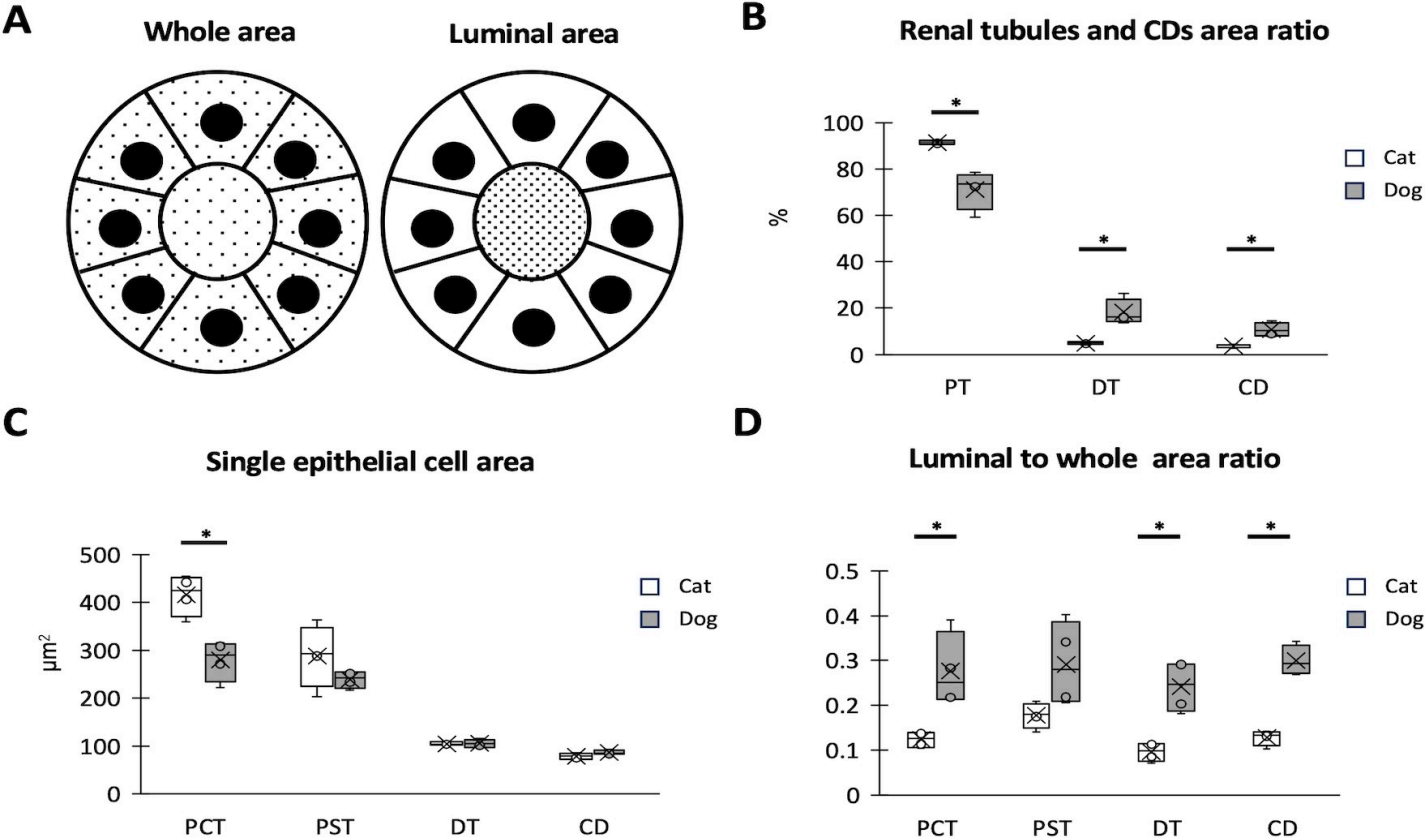
Histomorphometric characteristics of each renal tubule and collecting duct (CD) in adult cat and dog renal cortices. (A) Definition of whole and luminal areas in renal tubules and CDs. The dotted area represents the defined area. (B) Occupation ratios of proximal tubules (PTs), distal tubules (DTs), and CDs per unit area. (C) Size of single epithelial cells in the proximal convoluted tubules (PCTs), proximal straight tubules (PSTs), DTs, and CDs. (D) The ratio of the luminal area to the whole area in renal tubules and CDs. The box plot represents the minimum, first quartile, median, third quartile, and maximum (n >4). (n = 4). Significance with other species (Mann–Whitney *U*-test, * *P* <0.05).

For histomorphometry of the cortex, the whole and luminal areas of the renal tubules and CDs were defined (Fig 2A). PTs were observed more frequently than DTs or CDs in both species. However, cats showed significantly higher and lower values for PTs and others than dogs (Fig 2B). The single epithelial cell area was the largest in the PCTs of both species, but cats tended to have larger PTs than dogs, with a significant difference in PCTs (Fig 2C). Regarding the occupation ratio of the luminal area to the entire area, cats tended to show lower values than dogs in all examined tubules and CDs. Significant differences were detected in the PCTs, DTs, and CDs (Fig 2D).

### Fine segmentations of PTs in adult cat kidney

**Fig 3:**
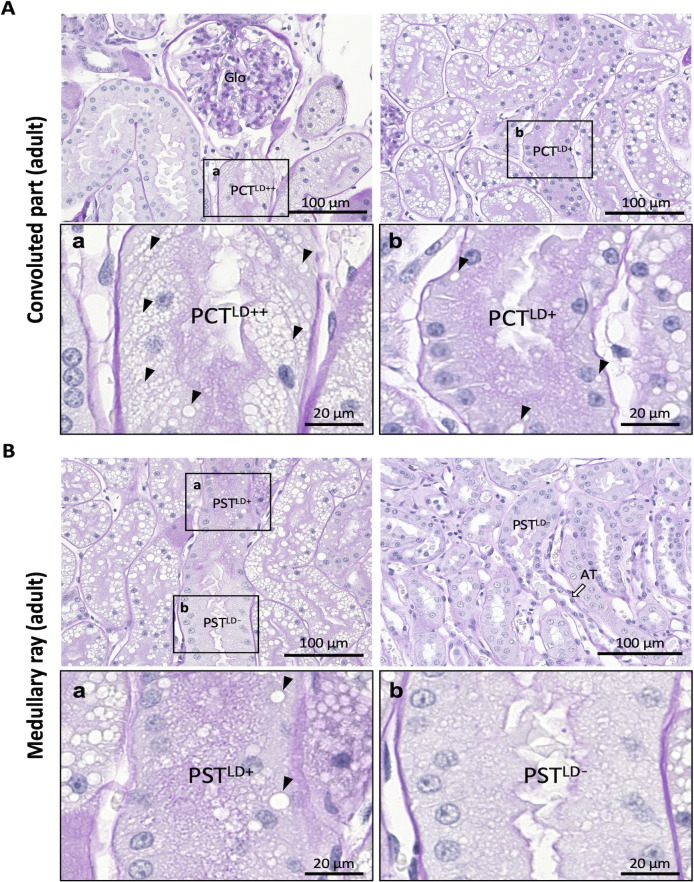
Fine segmentation of proximal tubules in adult cat kidney. (A) Proximal convoluted tubules (PCTs). Panels a and b show magnified squared areas in the upper panels. PCTs are divided into two segments: the segment containing abundant lipid droplets (LDs; arrowheads) in the cytoplasm (defined as PCT^LD++^, panel a) and the segment containing fewer droplets (defined as PCT^LD+^, panel b). PCT^LD++^ is close to the glomerulus (Glo), and PCT^LD+^ is far from Glo. (B) Proximal straight tubules (PSTs). Panels a and b show magnified squared areas in the upper panels. PSTs are divided into two segments: one containing LDs (arrowheads) in the cytoplasm (defined as PST^LD+^, panel a) and the other containing no LDs (defined as PST^LD-^, panel b). PST^LD+^ is close to PCTs, whereas PST^LD-^ is far from PCTs. Resolution of anatomical or histological images: 300 × 300 dpi. PAS-H staining. Bars = 20 μm (high magnification), 100 μm (low magnification). AT attenuated tubule.

The adult cat PTs were histologically observed in detail and divided into four segments (Fig 3). In the convoluted part, two types of PCT segments were observed: 1) PCT^LD++^ contained abundant LDs, which occupied >50% of the cytoplasmic area of epithelial cells and localized close to renal corpuscles (Fig 3A–a) and 2) PCT^LD+^ with a much smaller number of LDs, which occupied <50% of the cytoplasmic area of the cell and localized in the more distal part than PCT^LD++^ (Fig 3A–b). All the observed PCTs contained LDs in the epithelial cells.

In the medullary rays, two types of PST segments were observed: 1) PST^LD+^ contained visible LDs in the cytoplasm of epithelial cells (Fig 3Ba) and 2) PST^LD-^ contained almost no LDs and was localized in the more distal part of PST^LD+^ because it was connected to attenuated tubules (Fig 3B–b). Furthermore, the cytoplasm of PST^LD-^ was paler than that of PST^LD+^ in PAS-H staining.

### Fine segmentations of PTs in young cat kidneys

**Fig 4:**
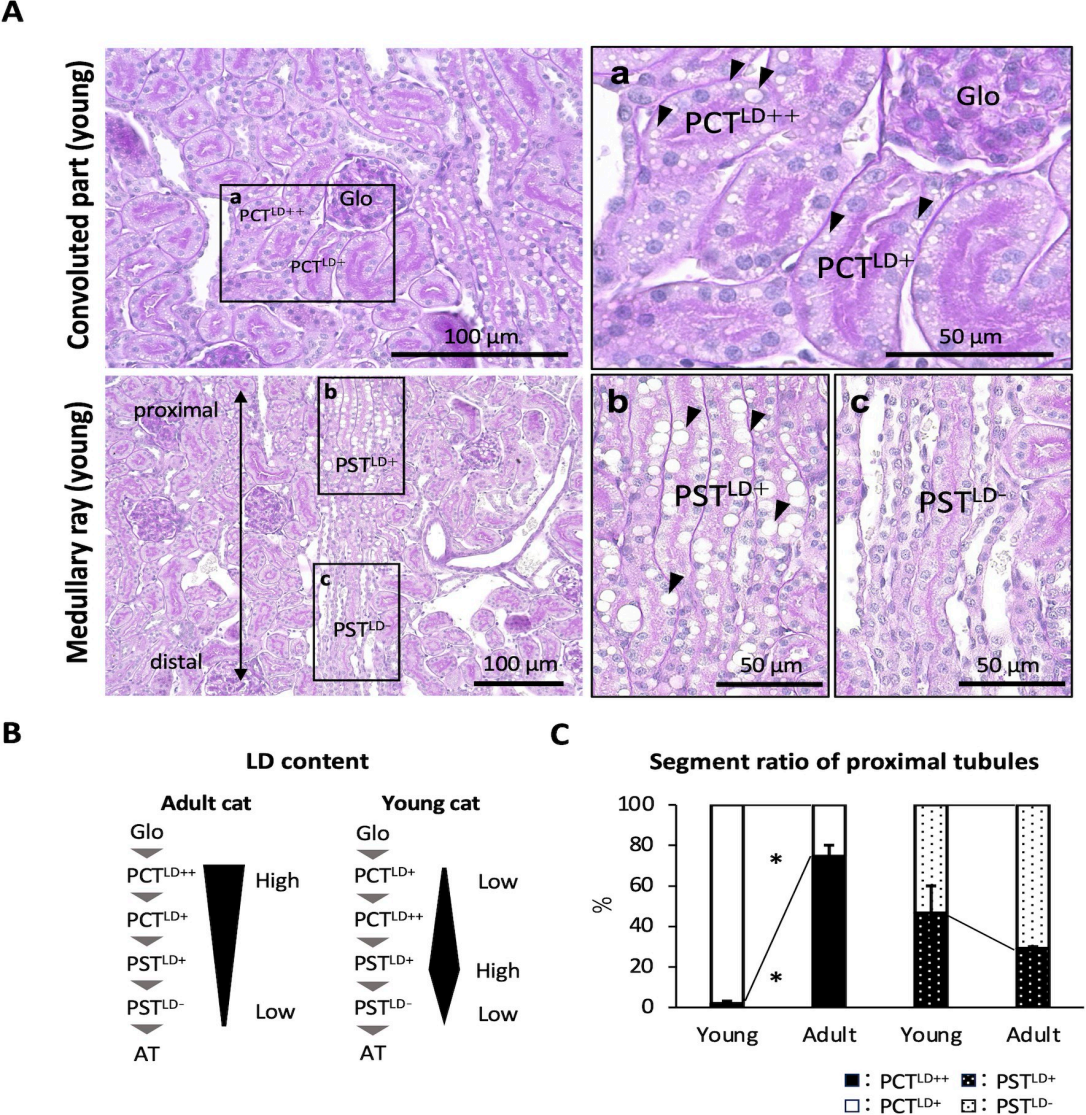
Fine segmentation of proximal tubules in young cat kidney. (A) Proximal convoluted tubules (PCTs) and proximal straight tubules (PSTs). Panels a, b, and c magnified the squared areas in the left panels. PCT^LD++^, PCT^LD+^, PST^LD+^, and PST^LD-^ are observed in adult cats; however, PCT^LD+^ is proximal to the glomerulus, and PCTLD++ is distal to the glomerulus (panel a). The PST^LD+^ is proximal to the PCTs, and PST^LD-^ is distal to the PCTs (panel b). Black arrowheads denote lipid droplets (LDs). PAS-H staining. Bars = 50 μm (high magnification), 100 μm (low magnification). Resolution of anatomical or histological images: 300 × 300 dpi. (B) Schematic diagram of LDs content in cat proximal tubules. (C) The ratio of each proximal tubular segment to total proximal tubules. Values = mean ± SE (n = 4). Significance with another age group (Mann–Whitney *U*-test, * *P* <0.05).

Four PT segments were also observed in young cats, but their lipid content tended to differ from that of adult cats. In the convoluted region, PCT^LD++^ was more distally localized than PCT^LD+^ (Fig 4A–a). In the medullary rays, PST^LD-^ was localized more distally than PST^LD+^ (Fig 4A–b and c). Fig 4B summarizes the fine segmentation of cat PTs. LDs were mainly localized to the epithelial cells of the proximal segments of PCTs and PSTs in adult and young cat kidneys, respectively. Furthermore, the number of PCT^LD++^ cats was significantly higher in adult cat PCTs than in young cats, but no significant age-related differences were detected in PSTs (Fig 4C).

### Glycosylation of renal tubules and CDs in cats and dogs

**Fig 5:**
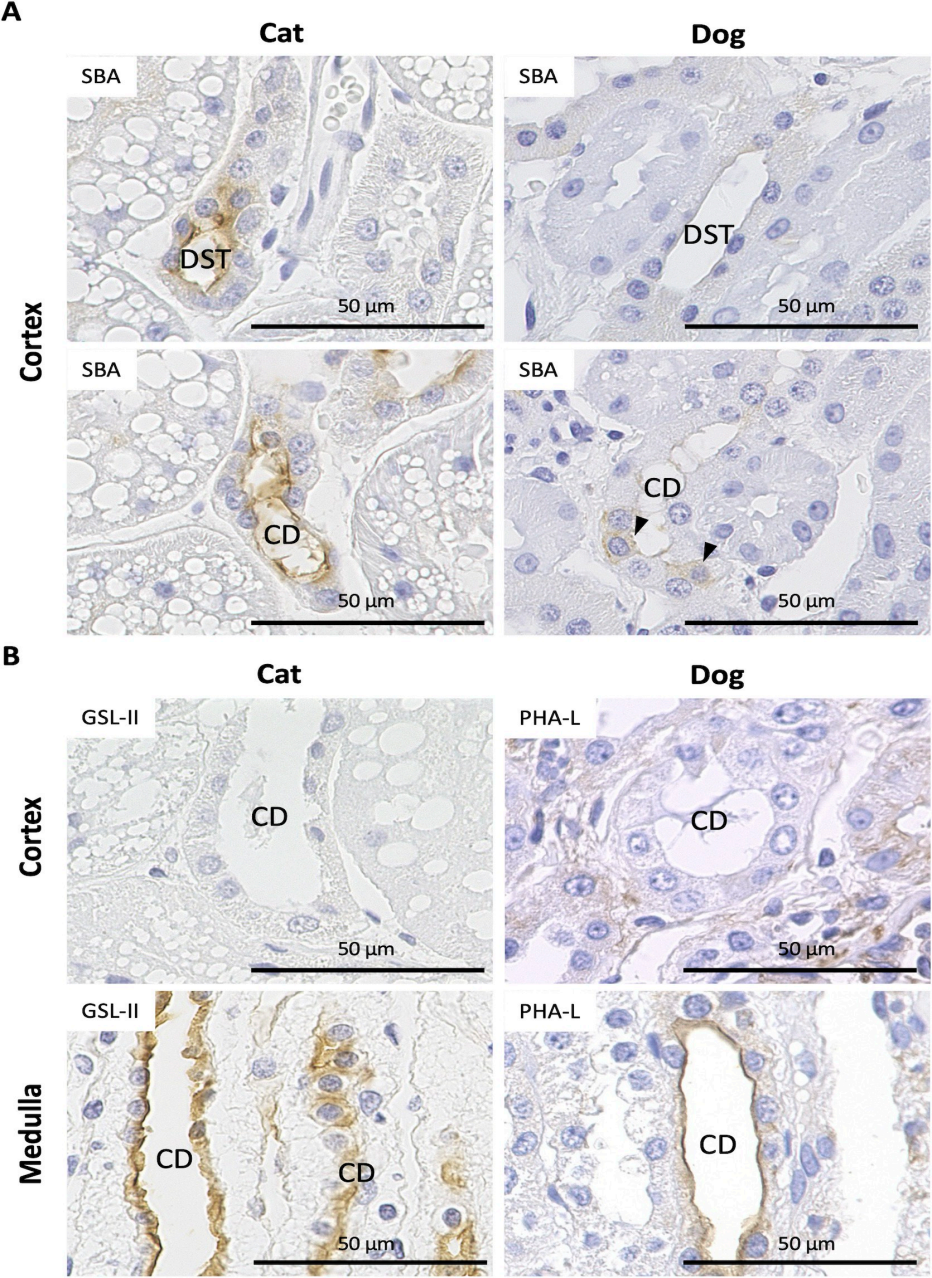
Representative images for lectin histochemistry of renal tubules and collecting ducts (CDs) in cats and dogs. (A) *Soybean agglutinin Glycine max* (soybean) seeds (SBA). In cats, positive reactions are observed in the apical portion of epithelial cells in the distal straight tubules (DSTs) and CDs. In dogs, these reactions are weak in DSTs; furthermore, several CD epithelial cells show SBA-positive reactions in the cytoplasm (arrowheads), though there are several negative cells. (B) *Griffonia simplicifolia* Ⅱ (GSL-Ⅱ) and *Phaseolus vulgaris* leukoagglutinin (PHA-L). Positive reactions for GSL-II and PHA-L are observed in the apical portion of the CD epithelium at the medulla but not in the cortex of cats and dogs, respectively. Bars = 50 μm.

Lectin histochemistry has been used to identify tubular segment-specific glycosylation in the kidneys of several species, including dogs and cats [4, 5]. To clarify the glycosylation manner of nephrons and CDs, we analyzed 21 lectin species. As shown in Table 2, the nephron- or CD-specific lectin-binding patterns differed between cats and dogs. Fig 5 shows representative staining images for lectin histochemistry, showing species- or segment-specific differences. *Soybean agglutinin Glycine max* (soybean) seed^+^ reactions in apical DSTs and CDs were observed in cats but were faint, weak, or heterogeneous among ducts in dogs (Fig 5A). For *Griffonia simplicifolia* II in cats, positive reactions were observed in the epithelial cells of the outer medullary CD but not in the cortical CD (Fig 5B). Similarly, positive reactions were observed in the epithelial cells of the outer medullary CD for PHA-L in dogs but not in those of the cortical CD (Fig 5B). For some lectins in cats, such as *S*. *japonica* and *G*. *simplicifolia* II, positive reactions were observed in the cytoplasm of epithelial cells in PST^LD-^ but not in PCT^LD++^, PCT^LD+^, and PST^LD+^ (Table 2). Thus, glycosylation patterns in kidneys differ among segments of PTs, other nephrons, CDs, and animal species.

**Table 2:** Summary of positive lectin histochemistry in the kidneys of cats and dogs.

Species	Structures	ConA	SBA	WGA	DBA	UEA-Ⅰ	RCA-Ⅰ	PNA	GSL-Ⅰ	PSA	LCA	PHA-E	PHA-L	SJA	s-WGA	GSL-Ⅱ	DSL	ECL	Jacalin	LEL	STL	VVA
**Cat**	**Renal corpuscle**	–	–	**Par-Cy, Pod-Cy**	–	–	**Par-Cy**	–	–	–	**Par-Cy, Pod-Cy**	–	**Par-Cy, Pod-Cy**	–	–	–	**Par-Cy, Pod-Cy**	–	**Par-Cy, Pod-Cy**	**Par-Cy, Pod-Cy**	**Par-Cy, Pod-Cy**	–
**Dog**							–	**Par-Cy**			–		–				–	–	**Par-Cy**	–	–	
**Cat**	**PCT** ^**LD++**^	–	E-BB	**E-Cy, E-BB**	–	–	**E-Cy,** E-BB	–	–	**E-Cy, E-BB**	**E-Cy, E-BB**	–	E-BB	–	–	–	**E-Cy, E-BB**	**E-BB**	**E-BB**	**E-Cy, E-BB**	**E-Cy, E-BB**	–
	**PCT** ^**LD+**^																					
**Dog**	**PCT**		–	E-BB			–			–	–		–				–		–	–	E-BB	
**Cat**	**PST** ^**LD+**^	–	E-BB	**E-Cy,** E-BB	–	–	**E-Cy,** E-BB	–	–	**E-Cy, E-BB**	**E-Cy, E-BB**	–	E-BB	–	–	–	**E-Cy, E-BB**	**E-BB**	**E-BB**	**E-Cy, E-BB**	**E-Cy, E-BB**	–
	**PST** ^**LD-**^		E-Cy, E-BB		E-Cy			E-Cy	E-Cy	E-Cy	E-Cy		E-Cy, E-BB	***E-Cy***		E-Cy		E-Cy, **E-BB**	E-Cy			E-Cy
**Dog**	**PST**		–	**E-BB**	–		–	–	–	–	–		–	–		–	–	**E-BB**	–	–	E-BB	–
**Cat**	**Attenuated tubule**	–	**E-Ap**	**E-Cy, E-Ap**	–	–	**E-Cy, E-Ap**	**E-Ap**	–	**E-Ap**	–	–	**E-Cy, E-Ap**	***E-Ap***	**E-Cy**	–	**E-Cy, E-Ap**	–	**E-Cy, E-Ap**	**E-Cy, E-Ap**	**E-Cy, E-Ap**	**E-Ap**
**Dog**				**E-Ap**			–	–		–			–	–	–		–		–	–	–	–
**Cat**	**Distal straight tubule**	–	**E-Ap**	**E-Cy, E-Ap**	–	–	**E-Cy, E-Ap**	**E-Ap**	–	–	–	–	**E-Ap**	–	–	–	**E-Cy, E-Ap**	**E-Ap**	**E-Ap**	**E-Cy, E-Ap**	**E-Cy, E-Ap**	–
**Dog**				**E-Ap**			–						–				–	–		**E-Ap**	–	
**Cat**	**Distal convoluted tubule**	–	**E-Ap**	E-Cy, E-Ap	–	–	**E-Cy,** E-Ap	***E-Ap***	–	–	–	–	**E-Ap**	–	**E-Ap**	–	**E-Cy, E-Ap**	**E-Ap**	**E-Ap**	**E-Cy, E-Ap**	**E-Cy, E-Ap**	**E-Ap**
**Dog**				**E-Ap**			–						–		–		–	–		**E-Ap**	–	–
**Cat**	**CCD**	–	**E-Ap**	**E-Cy, E-Ap**	–	–	**E-Cy, E-Ap**	**E-Cy, E-Ap**	–	**E-Ap**	**E-Ap**	–	**E-Ap**	–	–	–	**E-Cy, E-Ap**	–	**E-Cy, E-Ap**	**E-Cy, E-Ap**	**E-Cy, E-Ap**	**E-Ap**
**Dog**			***E-Cy*, *E-Ap***	**E-Ap**			–	**E-Ap**		–	–		–				–		**E-Ap**	**E-Ap**	–	–
**Cat**	**OMCD**	–	–	**E-Cy, E-Ap**	–	**E-Ap**	**E-Cy, E-Ap**	**E-Cy, E-Ap**	–	**E-Cy**	**E-Cy**	–	–	–	**E-Ap**	**E-Ap**	**E-Cy, E-Ap**	**E-Cy, E-Ap**	**E-Cy, E-Ap**	**E-Cy, E-Ap**	**E-Cy, E-Ap**	–
**Dog**			**E-Ap**	**E-Ap**	**E-Ap**	–	–	**E-Ap**		–	–		E-Ap		–	–	–	–	**E-Ap**	–	**E-Ap**	
**Cat**	**IMCD**	–	–	**E-Cy, E-Ap**	–	–	E-Cy, E-Ap	**E-Ap**	–	–	–	–	***E-Ap***	**E-Ap**	**E-Cy, E-Ap**	**E-Cy, E-Ap**	**E-Cy, E-Ap**	**E-Cy, E-Ap**	**E-Cy, E-Ap**	**E-Cy, E-Ap**	**E-Cy, E-Ap**	**E-Ap**
**Dog**				**E-Ap**	**E-Ap**		–	**E-Ap**					–	–	–	–	–	–	**E-Ap**	–	**E-Ap**	–
**Cat**	**Intestitium, capillary**	–	**E-Cy**	–	–	–	**E-Cy**	**E-Cy**	**E-Cy**	**E-Cy**	**E-Cy**	–	**E-Cy**	–	–	–	**E-Cy**	–	**E-Cy**	**E-Cy**	**E-Cy**	–
Dog								–				**E-Cy**					–					

PCT, proximal convoluted tubule; PST, proximal straight tubule; AT, attenuated tubule; DCT, distal convoluted tubule; CCD, cortical collecting duct; OMCD, outer medullary collecting duct; IMCD, inner medullary collecting duct; BV, blood vessel; Cap, capillary; SM, smooth muscle; CF, collagen fiber; BC, Bowman’s capsule; Cy, cytoplasm; BB, brush border; Ap, apical portion; −, negative. Bold, normal, and italics indicate strong, weak, and heterogeneous positive reactions, respectively.

### SGLT2 mRNA expression in the kidneys of cats and dogs

**Fig 6:**
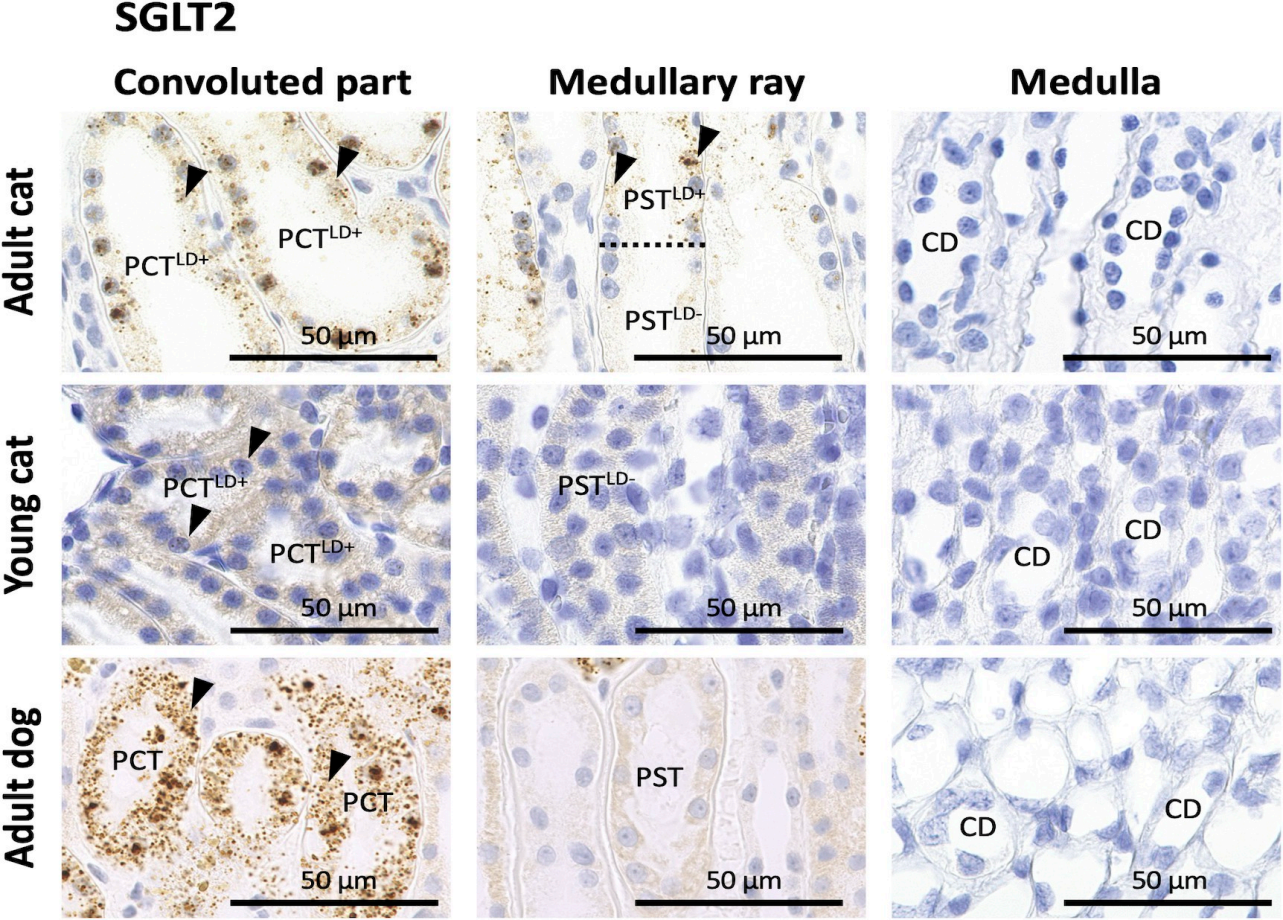
SGLT2 mRNA expression in the kidneys of cats and dogs. *SGLT2* location was analyzed using *in situ* hybridization (ISH) in adult cats, young cats, and adult dogs. *SGLT2-*positive (^+^) reactions (arrowheads) were observed in the cytoplasm or nuclei of epithelial cells of proximal convoluted tubules (PCTs) but not in those of proximal straight tubules (PSTs), distal tubules (DTs), or collecting ducts (CDs) in adult and young cats and adult dogs. In adult cats, *SGLT2*^+^ reactions were observed in the epithelial cells of PST^LD+^ but not in those of PST^LD-^ cats (the dotted line denotes the border between two segments). Resolution of anatomical or histological images: 300 × 300 dpi. Bars = 50 μm.

To characterize each renal tubule or CD segment in cats and dogs, we also evaluated the expression of *SGLT2*, which is mainly expressed in PCT in the human kidney [26] and is targeted as a therapeutic molecule for both CKD and diabetes [13]. In adult cat kidneys, positive reactions were observed in the cytoplasm or nuclei of epithelial cells in PCT^LD++^, PCT^LD+^, and PST^LD+^ but not in PST^LD-^, other nephron segments, or CDs. In young cat and adult dog kidneys, positive reactions were observed in the cytoplasm or nuclei of epithelial cells in PCTs but not in PSTs, other nephron segments, or CDs (Fig 6).

## Discussion

**Fig 7:**
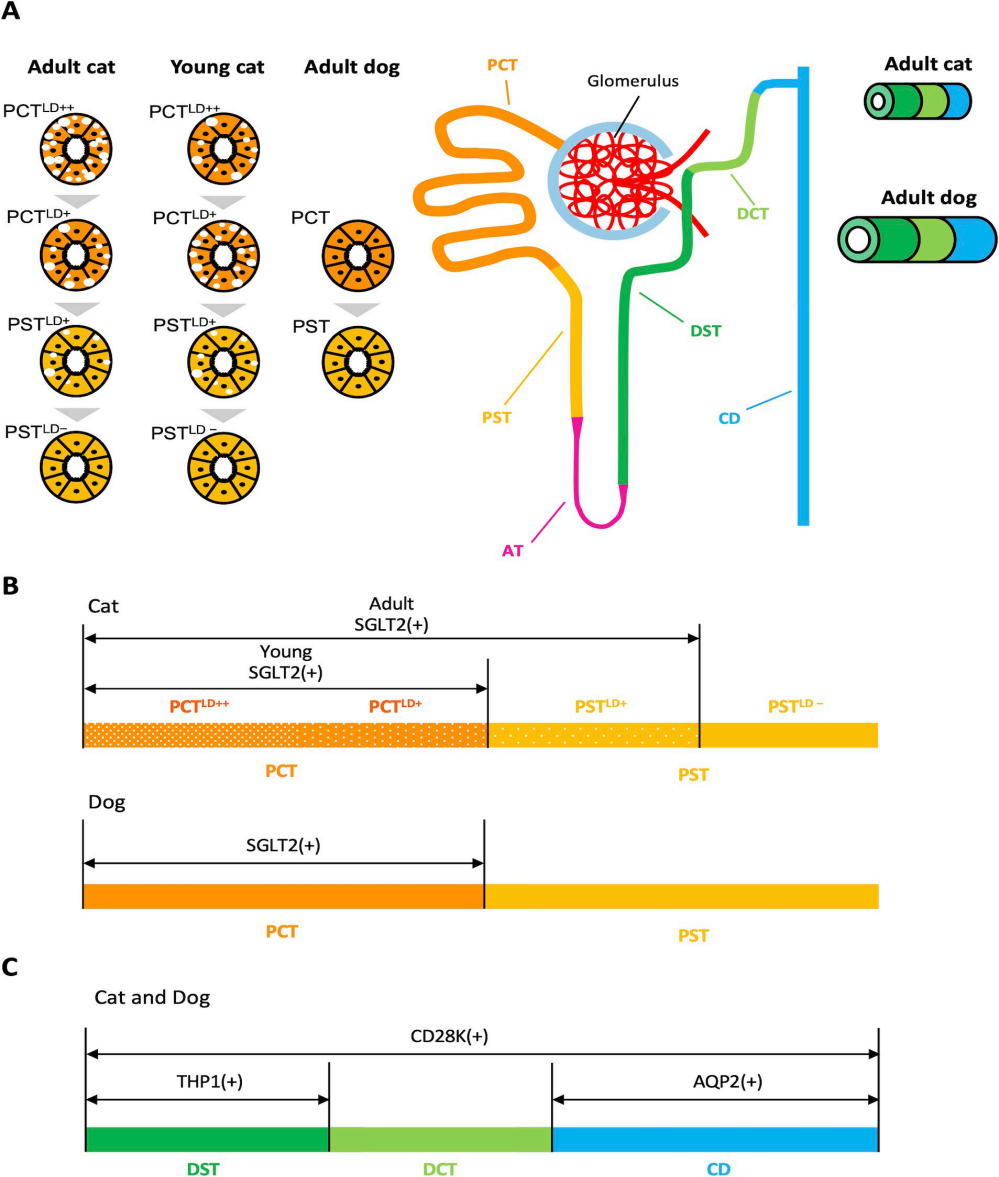
Schematic diagram of renal histology in cats and dogs. The upper schematic shows nephrons and collecting ducts (CDs) in adult cats. In cats, PTs are divided into four segments according to the lipid droplet (LDs) content in the cytoplasm of epithelial cells: proximal convoluted tubule (PCT)^LD++^, PCT^LD+^, proximal straight tubule (PST)^LD+^, and PST^LD-^ in that order from the glomerulus. Epithelial cells of PTs in cat and dog kidneys express mRNA of sodium-glucose cotransporter 2 (*SGLT2*), but those of PST^LD-^ in cats and dogs with PST do not. As shown in the lower schematic, the distal tubules (DTs) and CDs can be visualized by the differential expression of Tamm–Horsfall protein 1 (THP1), calbindin-D28K (CD28K), and aquaporin 2 (AQP2) in cats and dogs. The sizes and lumens of the DTs and CDs were smaller and narrower, respectively, in adult cats than in dogs (dialogue balloons).

Our results clarified the histological characteristics of cat and dog kidneys (Fig 7). In the renal cortex, the size of single epithelial cells and the occupation ratio of PTs were significantly larger in adult cats than in dogs. The occupation ratios of DTs and CDs, as visualized by the differential expression of THP1, CD28K, and AQP2, were lower in adult cats than in dogs. Furthermore, the lumens of PTs, DTs, and CDs were narrower in adult cats than in dogs. For the first time, cat PTs were shown to be divided into four segments according to the number of cytoplasmic LDs, and their appearance changed with age. Comprehensive lectin histochemistry revealed these two companion animals’ species- and segment-specific glycosylation patterns. We also measured the mRNA expression of *SGLT2*, a novel candidate molecule for human kidney disease therapy, in the PTs of cat and dog kidneys.

We identified four PT segments in adult cats according to the location and number of cytoplasmic LDs. Previous studies have reported three PT segments (S1, S2, and S3) in healthy cats based on the staining patterns for PAS and AQP1 localization: S1 in PCT with relatively pale cytoplasm and numerous cytoplasmic vacuoles, S2 in PCT with a magenta-colored cytoplasm and a few cytoplasmic vacuoles, and S3 in PST with cuboidal to columnar epithelial cells and scarce cytoplasmic vacuoles [2]. Similar to cat kidneys, rats and humans have S1 in PCT, characterized by greater cell height, a well-developed brush border, and basal infoldings; S2 in PCT with a less-developed brush border and basal infoldings than S1, and S3 in PST with few mitochondria and basal infoldings [8, 9]. The present study focused on cytoplasmic LDs of cat PTs, and we considered that PCT^LD++^ and PCT^LD+^ corresponded to S1 and S2, and PST^LD+^ and PST^LD-^ were S2 and S3, based on these previous criteria. These data reflect the PT segment-specific differences in epithelial histology, suggesting their functions differ between PT segments and animal species. Young cats also showed four PT segments; however, LDs were abundant in the PCTs and PSTs of adult and young cats, suggesting developmental changes in PT histomorphology related to lipid metabolism in the kidneys.

Cytoplasmic vacuolization in PT epithelial cells indicates that swollen mitochondria or multilamellar bodies appear depending on health or pathological conditions; however, vacuolar structures in felid PT epithelial cells are generally known as LDs [27, 28]. Mice injected with bovine serum albumin replete with free fatty acids showed severe tubulointerstitial injury characterized by PT damage [19]. LDs serve as energy deposits that segregate potentially toxic metabolites. Excess intracellular fatty acids in LDs can activate various stress signaling pathways, including inflammasome activation, and induce apoptosis [29–31]. Recently, lipids have received increasing attention because of their involvement in CKD induction and progression [32]. In contrast to lipid metabolism in humans, the primary cause of hepatic lipidosis in cats is a negative energy balance, usually caused by fasting and anorexia [33].

Previous studies have suggested that the LDs in cat kidneys contain triglycerides, phosphoglycerides, and steroids, particularly cholesterol, which are associated with sex hormones [34]. Although the present study investigated only intact male cats, LDs in the PTs increase following castration and estrus in both sexes [34]. Therefore, differences in the localization and number of LDs among PT segments, ages, and species may be affected by differences in lipid transport and generation. Felids, including cats, are highly susceptible to CKD and tubulointerstitial damage [16]. However, the relationship between the LDs in cats with PTs and their high susceptibility to CKD remains unclear. Therefore, a comprehensive analysis of the renal lipidome would be useful to clarify the biological significance of LDs and their relationship with CKD pathogenesis in cats.

A recent study showed that one of the important pathological characteristics often observed in AKI is renal tubular obstruction caused by debris from dead tubular epithelial cells and the subsequent proliferation of surviving epithelial cells in these structures [35–37]. In cat PTs, clearance of lumen-obstructing necrotic cell debris is inefficient because cat AIM does not dissociate from IgM during AKI [38]. Since the lumen size of PTs is relatively narrow in cats compared to dogs, this characteristic of cat PTs might contribute to the local obstruction of cell debris in pathological conditions. Furthermore, the lumens of the DTs and CDs were narrower than those of the PTs, and this tendency was more pronounced in cats than in dogs. Therefore, DTs and CDs may be more vulnerable to blockage by primary urine and cell debris than PTs, particularly in cats. THP1, a marker for DTs, is the main component of urinary casts [39], and THP1 may be as important as lumen-obstructing cell debris.

Glycans are expressed on the cell surface and play an important role in cell-to-cell communication [40]. Glycosylation enables glycoproteins to adopt diverse structures to perform various physiological functions [41]. Differences in glycosylation patterns should correlate with differences in glycosyltransferases, cell function, and pathological mechanisms of kidney diseases [42–45]. Protein O-GlcNAcylation is essential for renal lipolysis and ATP production during prolonged fasting [46]. To identify the glycosylation manner in cat and dog kidneys, we used 21 lectins, which are proteins with specific binding activity to sugar moieties on proteins or glycoproteins in animals [47]. Differences in the glycosylation manner reflects differences in cell functions and pathological mechanisms between carnivores. As representative cases, clear and heterogeneous positive reactions to *Soybean agglutinin Glycine max* (soybean) seeds were observed in apical cat DSTs and dog CDs. Although there have been no detailed reports regarding the functional significance of heterogeneous lectin binding, it indicates a single cell-specific histomorphology within a segment of nephrons or CDs.

Various nephron segment-specific molecules, such as those in the glucose transporter family, exhibit segment-specific functions. To date, our study is the first to show that *SGLT2* mRNA expression is the most important glucose transporter in the kidneys of cats and dogs, and the localization of its expression differed between the two carnivores. Recently, SGLT2 has been targeted for treating CKD in humans as it is a well-established molecular therapeutic target [13]. Characteristically, this correlated with LD disappearance in cat PT epithelial cells. Localization of *SGLT2-expressing* cells in PTs was also correlated with the disappearance of LDs, emphasizing the importance of analyzing both glucose and lipid metabolism to clarify the physiological and pathological characteristics of cat PTs. Therefore, data on SGLT2 expression in kidneys are important for developing future therapeutic strategies in the veterinary field.

In conclusion, this study clarified segment- or species-specific histology, such as lumen or tubule/duct sizes, cytoplasmic LD appearance in epithelial cells, and the expression of molecules, including SGLT2, in the renal tubules and CDs of cats and dogs. These findings are crucial for understanding species-specific characteristics of renal histomorphology and pathogenesis.

## Supporting information

S1 Table The list of antibodies used in this study. (PDF)

S2 Table The list of lectins used in this study. (PDF)
