# Supplementary material for: Species-specific histological characterizations of renal tubules and collecting ducts in the kidneys of cats and dogs
Source: PLoS One. 2024 Jul 3;19(7):e0306479. doi: 10.1371/journal.pone.0306479 (PMC11221681; doi:10.1371/journal.pone.0306479)
Supplement: S1 Table — (PDF) [file pone.0306479.s001.pdf]

Supplemental Table 1. The list of antibodies used in this study.

Purpose	Antigen	Host	Dilution	Source	Retrieval	Blocking
Primary	Tamm–Horsfall protein 1 (THP1)	Rabbit	1:400	Santa Cruz, Texas, USA	CB 115 °C 15 min	10 % goat serum
	Calbindin-D28K (CD28K)		1:1000	Proteintech, Tokyo, Japan	Tris-HCl 115 °C 15 min	
	Aquaporin 2 (AQP2)		1:800	Alpha Diagnostic Intl, Texas, USA		
Secondary	Rabbit-IgG (biotinylated)	Goat	Undiluted	SABRO(R) Kit, Nichirei, Tokyo, Japan	Not applicable	

CB, 10 mM citrate buffer (pH 6.0). Tris-HCl, 20 mM Tris-HCl (pH 9.0).
