# Supplementary material for: Species-specific histological characterizations of renal tubules and collecting ducts in the kidneys of cats and dogs
Source: PLoS One. 2024 Jul 3;19(7):e0306479. doi: 10.1371/journal.pone.0306479 (PMC11221681; doi:10.1371/journal.pone.0306479)
Supplement: S2 Table — (PDF) [file pone.0306479.s002.pdf]

Supplemental Table 2. The list of lectins used in this study.

Lectin	Abbreviation
Concanavalin A	ConA
Soybean agglutinin Glycine max (soybean) seeds	SBA
Wheat germ agglutin *Triticum vulgaris*	WGA
Dolichos biflorus agglutinin	DBA
Ulex europaeus agglutininI	UEA-I
Ricinus communis agglutinIseeds	RCA-I
Peanut agglutinin Arachis hypogaea peanuts	PNA
Griffonia simplicifolia lectinI	GSL-I
Pisum sativum agglutinin	PSA
Lens culinaris agglutinin	LCA
Phaseolus vulgaris Erythroagglutinin	PHA-E
Phaseolus vulgaris Leucoagglutinin	PHA-L
Sophora japonica	SJA
Succinylated Wheat Germ Agglutinin *Triticum vulgaris*	s-WGA
Griffonia simplicifolia II	GSL-II
Datura stramonium Lectin	DSL
Erythrina cristagalli Lectin	ECL
Jacalin *Artocarpus integrifolia* Seeds	Jacalin
Lycopersicon esculentum Lectin	LEL
Solanum tuberosum Lectin	STL
Vicia villosa Lectin	VVA
